# Elevated cAMP opposes (TNF-α)-induced loss in the barrier integrity of corneal endothelium

**Published:** 2010-09-02

**Authors:** Mahesh Shivanna, Sangly P. Srinivas

**Affiliations:** School of Optometry, Indiana University, Bloomington, IN

## Abstract

**Purpose:**

Elevated cyclic adenosine monophosphate (cAMP) enhances the barrier integrity of the corneal endothelium and thereby facilitates stromal hydration control, which is necessary for corneal transparency. This study investigates whether elevated cAMP is effective against the tumor necrosis factor-alpha (TNF-α)-induced loss of barrier integrity in monolayers of bovine corneal endothelial cells (BCEC).

**Methods:**

BCEC in primary culture were used for the study. Trans-endothelial electrical resistance (TER), a measure of barrier integrity, was determined by electrical cell-substrate impedance sensing. The changes were also ascertained by measuring paracellular permeability to fluorescein isothiocyanate (FITC)-dextran (10 kDa) across cells grown on porous culture inserts, and by immunofluorescence imaging of the apical junctional complex (AJC). The activation of p38 MAP kinase was assessed using western blotting.

**Results:**

Co-treatment with forskolin, which activates adenylate cyclase, and rolipram, which inhibits cAMP-dependent phosphodiesterase PDE4, reduced the TNF-α-induced increase in the flux of FITC-dextran. Similar co-treatment also prevented the TNF-α-induced disorganization of zona occludens-1 (ZO-1) and cadherins at the AJC. Co-treatment, as well pre-treatment, with forskolin plus rolipram prevented the TNF-α-induced decrease in TER. The influence of the agents was significant after 12 h of exposure to the cytokine. This effect was also mimicked by A2B agonists, adenosine and 5′-N-ethylcarboxamidoadenosine (NECA), which are known to mobilize cAMP in BCEC. Elevated cAMP also inhibited the cytokine-induced activation of p38 MAP kinase, and further blocked the disassembly of microtubules as well as the disruption of the PAMR (peri-junctional actomyosin ring) at the AJC.

**Conclusions:**

These results suggest that elevated cAMP opposes the TNF-α-induced loss in barrier integrity of the corneal endothelium. This effect follows inhibition of the cytokine-induced activation of p38 MAP kinase and its downstream signaling involved in the disruption of AJC and PAMR, as well as the disassembly of microtubules.

## Introduction

Corneal transparency depends on stromal deturgescence, which is maintained by the ‘fluid pump’ activity of its posterior monolayer, the endothelium [1-4]. A major threat to this hydration control is excessive fluid leakage into the stroma, which may occur upon failure of the barrier integrity associated with the endothelium. Situations involving barrier dysfunction are known to occur in response to inflammatory stress, such as during immune response secondary to allograft rejection [5-8], anterior uveitis [9], and iatrogenic injury [10]. Tumor necrosis factor-alpha (TNF-α) is a pro-inflammatory cytokine, the levels of which are significantly elevated in the aqueous humor during allograft rejection [7] and anterior uveitis [9]. A previous study by Watsky et al. [11] involving rabbit corneas mounted in vitro demonstrated that TNF-α breaks down the barrier integrity by disrupting the actin cytoskeleton. This response to TNF-α was opposed by a membrane-permeable analog of cyclic adenosine monophosphate (cAMP) [11]. However, the mechanisms underlying the disruption of the actin cytoskeleton by TNF-α and those responsible for influencing cAMP were not elucidated. It would be useful to know the molecular mechanisms involved in endothelial barrier dysfunction to develop pharmacological strategies to overcome stromal edema during transplantation failure and uveitis.

Adenosine and forskolin, agents known to elevate cAMP in the corneal endothelium, are known to promote stromal deturgescence in rabbits [12]. Specifically, it has been shown that these agents induce enhanced deswelling of preswollen rabbit corneas by enhancing the barrier integrity rather than by stimulating fluid transport [12,13]. In another study, rabbit corneal endothelium exposed to rolipram, a selective inhibitor of phosphodiesterase (isoform PDE4) [14], induced stromal thinning [15]. In our previous studies, we demonstrated that cAMP-induced myosin light chain (MLC) dephosphorylation blocked the thrombin- and histamine-induced breakdown of the barrier integrity in monolayers of bovine corneal endothelial cells (BCEC) [16-19]. In an analogous fashion, the breakdown of intercellular communication in bovine corneal endothelium in response to increased actomyosin contractions is also suppressed by elevated cAMP [20-26]. In addition to these effects of the second messenger on containing the events secondary to the direct increase of actomyosin contraction, our recent studies have focused on the influence of microtubule disassembly on the disruption of the actin cytoskeleton [27]. Thus, nocadazole-induced microtubule disassembly and the associated increase in actomyosin contraction could also be inhibited by elevated cAMP [27]. In a more recent study, we demonstrated that TNF-α induces microtubule disassembly and thereby contributes to barrier failure in BCEC [28]. In a subsequent study, we demonstrated that this TNF-α response is concomitant with the activation of p38 MAP kinase [29]. In this study, we investigated the influence of elevated cAMP on the (TNF-α)-induced barrier dysfunction in BCEC using trans-endothelial electrical resistance (TER) as a principal measure of barrier integrity. Our results show that agents that elevate cAMP also oppose (TNF-α)-induced p38 MAP kinase activation and thereby the loss of barrier function.

## Methods

### Materials

TNF-α (biologic activity of 2×10^7^ U/mg; endotoxin free), forskolin, adenosine, 5′-N-ethylcarboxamidoadenosine (NECA), rolipram, pan-cadherin antibody, α-tubulin antibody, and FITC-dextran were purchased from Sigma Aldrich (St. Louis, MO). Texas Red-conjugated phalloidin, goat anti-mouse Alexa-488, and anti-fade agent were purchased from Molecular Probes (Eugene, OR). The ZO-1 antibody was procured from Zymed (Long Island, NY). Electrodes (8W10E+) for TER measurements were purchased from Applied Biophysics, Inc. (Troy, NY). Tissue culture inserts (0.2 μm pore size) were obtained from Nunc (Naperville, IL). The enhanced chemiluminescence kit for western blotting analysis was obtained from Thermo Scientific (Rockford, IL).

### Culture of bovine corneal endothelial cells

Primary cultures of BCEC from fresh eyes were established as described previously [16-19]. The growth medium contained Dulbecco’s Modified Eagle's Medium (DMEM), supplemented with 10% bovine calf serum and an antibiotic-antimycotic mixture (penicillin, 100 U/ml, streptomycin 100 μg/ml, and amphotericin-B 0.25 μg/ml). Cells were cultured at 37 °C in a humidified atmosphere containing 5% CO_2_. The medium was replaced every 2–3 days. Cells of the first and second passages were harvested and seeded onto Petri dishes and glass coverslips and allowed to grow to confluence for 3–4 days before use. Cell culture supplies were from Invitrogen (Long Island, NY).

### Immunocytochemistry

The components of the cytoskeleton and apical junctional complex (AJC) were stained using an immunocytochemistry protocol, as described previously [27-31]. Cells grown on coverslips were washed with phosphate buffer saline (PBS) after the desired drug treatment, fixed with 3.7% paraformaldehyde and permeabilized using 0.2% Triton X-100 for 5 min. This was followed by staining for F-actin using phalloidin conjugated to Texas Red (1:1,000) for 45–60 min at room temperature (RT). Cells were stained for microtubules, zona occludens-1 (ZO-1), and pan-cadherin by fixing the cells, followed by permeabilization with 0.01% saponin in PBS. The cells were exposed to a blocking buffer for 45 min, and then incubated with the antibody for α-tubulin (1:1,000)/ZO-1 (1:25)/pan-cadherin (1:1,000) in a mixture of 0.01% saponin in PBS-goat serum (1:1) overnight at 4 °C. This was followed by washing and incubation with a secondary antibody (goat anti-mouse IgG Alexa Fluor 488 at 1:1,000). Stained cells were mounted using anti-fade medium containing 4',6-diamidino-2-phenylindole (DAPI), and then visualized using an epifluorescence microscope equipped with a 60× oil immersion objective and 1.2 NA (Nikon, Tokyo, Japan).

### Trans-endothelial electrical resistance (TER)

The TER was measured using the principle of electric cell-substrate impedance sensing using a commercial device (ECIS 1600R; Applied Biophysics, Inc., Troy, NY), as described previously [8,27-31]. Cells were seeded at a density of 5×10^5^ cells/ml on gold electrodes and grown to confluence. After the monolayers showed steady resistance values, they were exposed to desired agents after at least 1 h exposure to serum-free medium. The resistive portion of the measured impedance (TER), normalized to its initial value, was monitored continuously and taken as a measure of barrier integrity.

### Permeability to FITC-dextran

The changes in permeability to FITC-dextran were determined by a protocol, as described previously [27-31]. Cells were grown on 0.2 μm pore-size collagen IV (1 mg/mL)-coated tissue culture inserts (Nunc^™^; Fisher Scientific, Pittsburgh, PA) to confluence. The monolayers were then serum-starved for 1 h and either left untreated or exposed in triplicate to desired agents. Following treatment, FITC-dextran (10 kDa), dissolved in Ringers, was introduced in the apical compartment at a concentration of 0.4 μg/ml. After incubation at 37 °C for 2 h, samples were taken from the basolateral chamber for fluorescence measurements. Fluorescence was measured using excitation at 492 nm and the emission was collected at 520 nm.

### Western blot analysis

Confluent monolayers were serum-starved overnight before treatment with desired agents. The cells were then lysed with NP-40 lysis buffer (Invitrogen, Carlsbad, CA) containing protease inhibitor cocktail (Sigma Aldrich, St. Louis, MO) and 5× reducing sample buffer (Thermo Scientific, Rockford, IL). The lysates were transferred to a microcentrifuge tube kept on ice, followed by sonication for 10–15 s. The lysates were centrifuged at 14,000× g for 10 min followed by heating at 95–100 °C for 5 min. The samples were cooled to RT, centrifuged, and resolved by electrophoresis. This was followed by transfer onto a nitrocellulose membrane and incubation with the primary antibody (anti-phospho p38 MAP kinase; 1:1,000 dilution) overnight at 4 °C. The blots were washed with wash buffer, followed by incubation with HRP-conjugated secondary antibody for 1 h at RT. The blots were visualized using an enhanced chemiluminescence kit (Thermo Scientific, Rockford, IL). After visualization, the blots were stripped of phospho-p38 antibody and probed for total p38 MAP kinase with the anti-p38 MAP kinase antibody (1:1,000 dilution).

### Statistical analysis

Statistical comparisons were made using one-way ANOVA (ANOVA) with Bonferroni’s post-test analysis using GraphPad Prism software (Version 5.0; GraphPad Software, Inc., San Diego, CA). A value of p<0.05 was considered statistically significant. Data are expressed as mean ±SEM. The “n” represents the number of independent experiments.

## Results

### Effect of elevated cAMP on paracellular permeability

**Figure 1:**
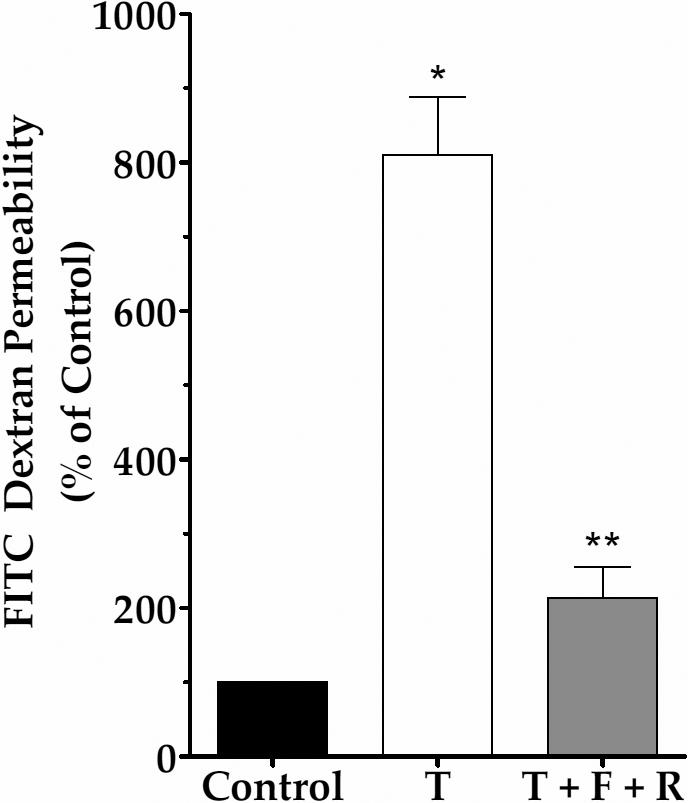
Effect of elevated cAMP on barrier integrity. Changes in paracellular permeability in response to TNF-α (20 ng/ml; T) with or without co-treatment with 10 µM forskolin (F) and 50 µM rolipram (R) were determined by quantifying the flux of FITC-dextran (10 kDa) across cells grown on porous culture inserts. Treatment with TNF-α for 20 h significantly increased the flux of dextran in comparison to untreated cells (Control). Co-treatment with F and R [i.e., TNF-α+F+R)] significantly attenuates the (TNF-α)-induced increase in permeability. Each bar represents mean±SEM of eight independent trials; * and ** denote p<0.001 when comparing the TNF-α-treated group with the control group and the (TNF-α+F+R)-treated group with the TNF-α-treated group, respectively.

We first examined the influence of cAMP on (TNF-α)-induced increase in the paracellular permeability to FITC-dextran. Exposure to 20 ng/ml of TNF-α for 20 h in the apical chamber increased the flux of FITC-dextran by >8 fold when compared to untreated cells (Figure 1). However, co-treatment with 10 μM forskolin and 50 μM rolipram opposed the cytokine-induced increase in permeability.

### Effect of elevated cAMP on the integrity of apical junctions

**Figure 2:**
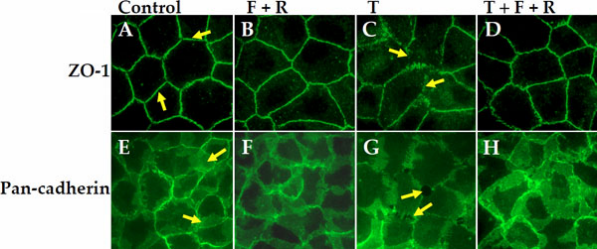
Effect of elevated cAMP on the distribution of ZO-1 and cadherins. Cells were treated with TNF-α (T) for 6 h and where indicated were co-treated with 10 µM forskolin (F) and 50 µM rolipram (R). **A**-**D**: Distribution of ZO-1. In untreated cells (Control, **A**), the ZO-1 is contiguous at the cell-cell border (shown by arrows). It is undisturbed by F and R (**B**). Exposure to TNF-α (**C**) induced dispersion of ZO-1 (shown by arrows), which was opposed by co-treatment with F and R (**D**). **E**-**H**: Pan-cadherins at the focal plane of AJC. In untreated cells (Control, **E**), localization of cadherins at the region of cell-cell contacts is intense (shown by arrows). Upon treatment with F and R (**F**), the distribution of cadherins is unaltered. Exposure to TNF-α (**G**) resulted in reduced intensity of cadherins at the cell border as well as sporadic disengagement (shown by arrows). Co-treatment with F and R (**H**) prevented the TNF-α response. All the images shown are representative of at least three independent experiments.

The influence of TNF-α on the integrity of tight junctions (TJs) and adherence junctions (AJs) was examined by immunostaining ZO-1 and cadherins as markers, respectively [32,33]. In untreated cells, the distribution of ZO-1 and cadherins is contiguous at the cell-cell borders, indicating stable TJs and AJs (Figure 2A,E, respectively). Upon treatment with forskolin and rolipram alone, the distribution of ZO-1 and cadherins was undisturbed, similar to that of untreated cells (Figure 2B,F, respectively). Co-treatment with forskolin and rolipram opposed the (TNF-α)-induced dislocation of ZO-1 and the redistribution of cadherins (Figure 2C versus Figure 2D; also Figure 2G versus Figure 2H, respectively). These findings are consistent with the effect of elevated cAMP on the (TNF-α)-induced increase in FITC-dextran permeability (Figure 1).

### Effect of elevated cAMP on TER dynamics

**Figure 3:**
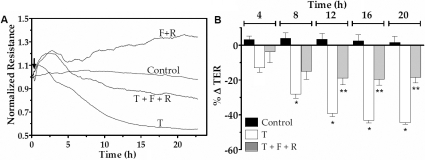
Effect of forskolin plus rolipram pre-treatment on TER response. The changes in TER were measured in response to TNF-α (T) with or without pre-treatment with 10 µM forskolin (F) and 50 µM rolipram (R) for 30 min. **A**: Typical responses. **B**: Summary of six independent experiments. The % reduction in TER induced by the cytokine is significant when compared to control at >8 h. Pre-treatment with F and R opposes the (TNF-α)-induced reduction in TER after 12 h of exposure. * and ** denotes p<0.001 when comparing the TNF-α-treated group with the control group and the (TNF-α+F+R) -treated group with the TNF-α-treated group, respectively.

**Figure 4:**
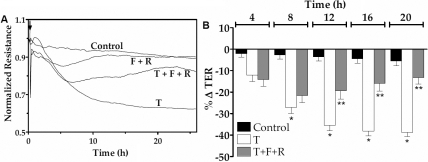
Effect of forskolin and rolipram co-treatment on TER dynamics. The changes in TER were measured in response to TNF-α (T) with or without co-treatment with 10 µM forskolin (F) and 50 µM rolipram (R). **A**: Typical responses. **B**: Summary of six independent experiments. The % reduction in TER induced by the cytokine is significant when compared to control at >8 h. Co-treatment with F and R opposes the (TNF-α)-induced reduction in TER after 12 h of exposure. * and ** denotes p<0.001 when comparing the TNF-α-treated group with the control group and the (TNF-α+F+R)-treated group with the TNF-α-treated group, respectively.

**Figure 5:**
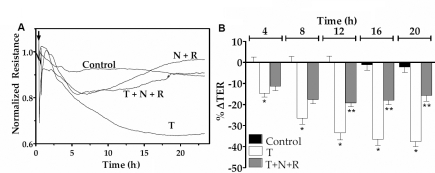
Effect of NECA on TER dynamics. The changes in TER were measured in response to TNF-α (T) with or without co-treatment with 20 µM NECA (N) and 50 µM rolipram (R). **A**: Typical responses. **B**: Summary of six independent experiments. The % reduction in TER induced by the cytokine is significant when compared to control at >8 h. Co-treatment with N and R opposes the TNF-α-induced reduction in TER after 12 h of exposure. * and ** denote p<0.001 when comparing the TNF-α-treated group with the control group and the (TNF-α+N+R)-treated group with the TNF-α-treated group, respectively.

**Figure 6:**
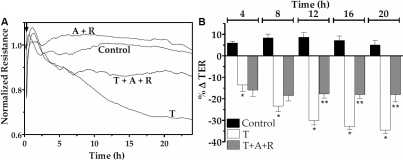
Effect of adenosine on TER dynamics. The changes in TER were measured in response to TNF-α (T) with or without co-treatment with 200 µM adenosine (A) and 50 µM rolipram (R). **A**: Typical responses. **B**: Summary of six independent experiments. The percent reduction in TER induced by the cytokine is significant when compared to control at >8 h. Co-treatment with A and R opposes the TNF-α-induced reduction in TER after 12 h of exposure. * and ** denote p<0.001 when comparing the TNF-α-treated group with the control group and the (TNF-α+A+R)-treated group with the TNF-α-treated group, respectively.

To assess the influence of elevated cAMP on TNF-α response at a higher temporal resolution, we reexamined the barrier integrity in terms of TER, as measured using the principle of electrical cell-substrate impedance sensing [34]. As demonstrated in our previous studies [28,29], exposure to 20 ng/ml of TNF-α induced a decline in TER after a delay of about 2.5 h for more than 20 h. However, pre-treatment with forskolin and rolipram for 30 min opposed the reduction in TER (Figure 3A). Data from independent experiments similar to that shown in Figure 3A are summarized in terms of percent reduction in TER at 4 h intervals after exposure to the cytokine (Figure 3B). It should be noted that the pre-treatment with forskolin and rolipram attenuated the (TNF-α)-induced decline in TER by >2 fold after 12 h. Similarly, as shown in Figure 4, co-treatment of the same drugs with TNF-α also opposed the TNF-α response by about twofold. We also examined whether other agents known to elevate cAMP can mimic the effect of forskolin. Co-treatment of NECA, (a potent A2B agonist) or adenosine (an endogenous A2B agonist) with rolipram also attenuated the (TNF-α)-induced decline in TER by about twofold (Figure 5A and Figure 6A, respectively). As shown in Figure 5B and Figure 6B, the effects of both NECA and adenosine are significant after 12 h. These results demonstrate that elevated cAMP opposes the loss of integrity of the endothelial barrier induced by TNF-α.

### Effect of elevated cAMP on the p38 MAP kinase activation

**Figure 7:**
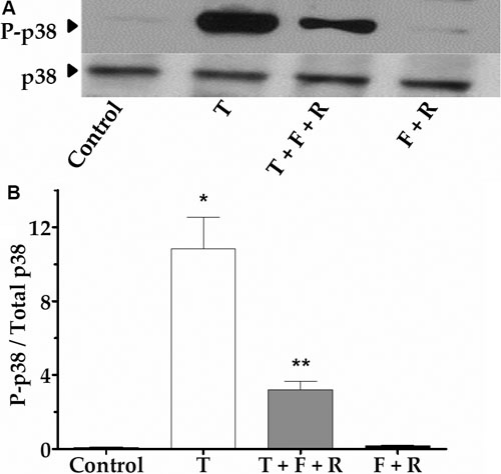
Effect of elevated cAMP on p38 MAP kinase activation. p38 MAP kinase activation following exposure to 20 ng/ml TNF-α (T) for 10 min with or without pre-treatment with 10 µM forskolin (F) and 50 µM rolipram (R) for 30 min was confirmed by immunoblotting using both anti-phospho-specific p38 MAP kinase antibody and anti-p38 MAP kinase antibody. **A**: TNF-α-induced phosphorylation of p38 MAP kinase following 10 min of exposure. However, pre-treatment with forskolin (F) and rolipram (R) suppressed p38 MAP kinase phosphorylation induced by TNF-α (T). **B**: Bar graph of the densitometric analysis of three independent experiments similar to that shown in **A**. The ratio of phosphorylated to total p38 MAP kinase following TNF-α stimulation is significantly increased when compared to untreated cells. Pre-treatment with F and R followed by stimulation with TNF-α significantly decreases the ratio. * and ** denotes p<0.001 when comparing the TNF-α-treated group with the control group and the (TNF-α+F+R)-treated group with the TNF-α-treated group, respectively.

Recently, we showed that p38 MAP kinase is activated in response to TNF-α [29]. The activation peaked at 10 min and its inhibition was found to effectively oppose the cytokine-induced microtubule disassembly, as well as loss of barrier function in BCEC [28]. Thus, we examined p38 MAP kinase activation upon exposure to TNF-α with or without forskolin and rolipram. As shown in Figure 7A, exposure to 20 ng/ml of TNF-α for 10 min induced p38 MAP kinase phosphorylation. However, upon pretreatment with forskolin and rolipram for 30 min, (TNF-α)-induced phosphorylation was suppressed. The bar graph of the densitometric analysis of similar experiments is shown in Figure 7B. It is clear that elevated cAMP attenuates (TNF-α)-induced p38 MAP kinase activation by about threefold at 10 min.

### Effect of elevated cAMP on the cytoskeleton

**Figure 8:**
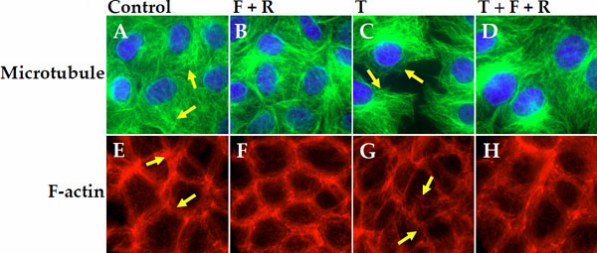
Effect of elevated cAMP on the organization of microtubules and PAMR. Cells were treated with 20 ng/ml TNF-α (T) for 6 h and where indicated were co-treated with 10 µM forskolin (F) and 50 µM rolipram (R). **A**-**D**: Organization of microtubules. In untreated cells (Control, **A**), microtubules exist as characteristic fibrillary extensions from around the nucleus to the cell periphery (shown by arrows). It is undisturbed by F and R (**B**). Treatment with TNF-α (**C**) induced microtubule disassembly characterized by the loss of as well as condensation of fibrillary extensions (shown by arrows), which was opposed by co-treatment with F and R (**D**). **E**-**H**: Organization of PAMR. In untreated cells (Control, **E**), the characteristic organization of cortical actin with intact PAMR is observed (shown by arrows). It is undisturbed by F and R (**F**). Treatment with TNF-α induced disruption of PAMR (**G**) (shown by arrows), which was opposed by co-treatment with F and R (**H**). All the images shown are representative of at least three independent experiments.

In our previous study [29], we observed that (TNF-α)-induced p38 MAP kinase activation underlies the disruption of the peri-junctional actomyosin ring (PAMR) as well as the disassembly of the microtubules. Therefore, we examined whether these responses are also blocked by elevated cAMP. Figure 8 shows the influence of TNF-α on the organization of microtubules and PAMR with and without co-treatment with forskolin and rolipram. In untreated cells (Figure 8A), microtubules extend from around the nucleus toward the cell periphery as fibrillary structures. Treatment with 20 ng/ml TNF-α for 6 h led to loss of the fibrillary extensions (Figure 8C). This loss is suppressed when cells are co-treated with forskolin and rolipram (Figure 8D). Similarly, as shown in Figure 8G, the (TNF-α)-induced disruption of the PAMR is also opposed when cells are co-treated with forskolin and rolipram (Figure 8H). Upon treatment with forskolin with rolipram, the organization of microtubules (Figure 8B) as well as PAMR (Figure 8F) was unaltered and similar to control. These results indicate that elevated cAMP opposes the (TNF-α)-induced disruption of PAMR as well as microtubule disassembly.

## Discussion

Corneal endothelial dysfunction due to aging, iatrogenic injury, Fuch’s dystrophy, and inflammation is known to induce stromal edema leading to loss of acute vision [3,8]. Currently, there are no pharmacological interventions to overcome the endothelial dysfunction, and the only recourse is corneal transplantation. However, inflammatory stress during transplantation failure and uveitis is of concern [7,8,11,35]. In this context, we investigated the role of TNF-α, a prominent pro-inflammatory cytokine thought to play a major role in corneal endothelial dysfunction [7,8,11,35]. The current study focused on the role of barrier dysfunction induced by the cytokine and its modulation by cAMP. Our major finding is that elevated cAMP attenuates the (TNF-α)-induced barrier dysfunction by inhibiting p38 MAP kinase activation. Thus, the findings of this study suggest it is possible to rescue corneas undergoing transplantation failure or challenged by anterior uveitis using topical agents involving cAMP-dependent PDE inhibitors and/or A2B agonists, which can elevate cAMP in the corneal endothelium.

In our first study on TNF-α [28], we demonstrated that the cytokine induces microtubule disassembly concomitant with loss of barrier integrity in BCEC monolayers. In a subsequent study, we further showed that the cytokine effect involves activation of p38 MAP kinase, which is a stress-activated kinase [29]. Inhibition of p38 MAP kinase by its selective inhibitor SB-203580 markedly attenuated microtubule disassembly. This is consistent with the loss of barrier integrity in response to nocadazole-induced microtubule disassembly, as shown in our previous study with BCEC [27]. The latter study [27] also demonstrated that the loss of barrier integrity induced by microtubule disassembly could be attenuated by co-treatment with forskolin and rolipram. Therefore, in this study, we began to investigate the effect of elevated cAMP on the response to TNF-α.

In our first series of experiments, the barrier integrity was assessed by following the flux of FITC-dextran. A nearly eightfold increase in permeability to the dextran was reduced to only twofold when cells were co-treated with forskolin and rolipram (Figure 1). Since (TNF-α)-induced loss in barrier integrity is not due to apoptosis, as shown in our previous study [29], the observed effect of cAMP cannot be attributed to cell survival. The effect of cAMP against the loss in barrier integrity is also revealed by immunofluorescence of the AJC complex (Figure 2). Specifically, the discontinuity in ZO-1 distribution at the cell-cell border in response to TNF-α (Figure 2C) is not noticeable upon treatment with forskolin and rolipram (Figure 2D). This is a clear indication of stable TJs. The loss of AJs, evident because of the disassociation of cadherins (Figure 2G) at the cell-cell border in response to the cytokine, is also eliminated by elevated cAMP (Figure 2H). Thus, both TJs and AJs, which are disrupted in response to TNF-α, remain intact with elevated cAMP.

As shown in Figure 3A, Figure 4A, Figure 5A, and Figure 6A, TNF-α produced a steady decline in TER as expected. When cells were co-treated with forskolin, NECA, or adenosine, the decline in TER was significantly attenuated. The statistical summary in Figure 3B, Figure 4B, Figure 5B, and Figure 6B gives clear evidence for the influence of cAMP-elevating agents on the response to TNF-α. Specifically, each of the agents reduced the loss in TER, and the reduction became statistically significant (p<0.001) after 12 h of exposure to the cytokine. The apparent efficacy of NECA and adenosine when compared to that of forskolin is consistent with the reported activity of A2B receptors, which are coupled to Gs G protein, in the corneal endothelium [16,18,36]. Previously, we showed that adenosine enhances intracellular cAMP by 11-fold when compared to untreated cells [16]. Furthermore, both NECA and adenosine increased TER in untreated cells (Figure 5A and Figure 6A). This is consistent with our previous measurements of impedance with cells grown on porous filters [18]. Taken together, our findings in Figure 1, Figure 2, Figure 3, Figure 4, Figure 5, and Figure 6 confirm that cAMP-elevating agents oppose the loss of barrier integrity in response to TNF-α.

In our previous studies [28,29], we observed significant influence of the cytokine on the actin cytoskeleton, similar to what was observed by Watsky et al. [11] in rabbit corneal endothelial cells. Specifically, we found that the cytokine induces disassembly of microtubules [28], which in and of itself is able to induce disruption of the PAMR, as shown by our nocodazole study [27]. All these changes were opposed by the inhibition of p38 MAP kinase [29]. As a distinct pool of F-actin at the AJC, the PAMR is structurally and functionally coupled to TJs and AJs [8]. Upon excessive actomyosin contraction of the PAMR, the centripetal force generated around AJC reduces cell-cell tethering and this opposes the stable interaction of trans-membrane domains of the TJs and AJs [8,30]. In light of these observations, we examined whether the influence of cAMP-elevating agents also manifested at the level of the cytoskeleton. In all our experiments, in agreement with the containment of loss of barrier integrity, forskolin and rolipram conferred protection against the (TNF-α)-induced microtubule disassembly and disruption of the PAMR (Figure 8).

Although (TNF-α)-induced barrier dysfunction has been widely examined [11,37,38], the ability of cAMP-elevating agents to counter the influence of the cytokine has not received much attention [39,40]. One can find several reasons, however, for employing agents that elevate cAMP to oppose the response to TNF-α. First, the second messenger is tightly coupled to the regulation of the actin cytoskeleton by cross-talk between the RhoA-Rho kinase axis as well as Rac1-dependent mechanisms [41-43]. Second, TNF-α has been shown to elicit upregulation of cAMP-dependent phosphodiesterases [40]. Although the RhoA-Rho kinase axis is implicated in TNF-α signaling [44], there are no reports showing the efficacy of Rho kinase inhibitors against the loss of barrier integrity induced by the cytokine. Thus, mechanisms involved in the disruption of the actin cytoskeleton appear to be significantly influenced by pathways independent of the RhoA-Rho kinase axis.

The other major mechanisms implicated in the response to TNF-α include activation of ROS/RNS (reactive oxygen/nitrogen species) [45-47], stress kinases [38,48], microtubule disassembly [38,49], and transcriptional activation of genes that might influence the actin cytoskeleton, including myosin light chain kinase (MLCK) [44,50] and heat shock protein 27 (Hsp27) [51]. As previously noted, we earlier demonstrated a major role for p38 MAP kinase and microtubule disassembly in the response to TNF-α [28,29]. Although the underlying molecular mechanisms are unknown, it appears that the microtubule disassembly follows p38 MAP kinase activation [52,53]. Thus, inhibition of the kinase attenuated the cytokine-induced microtubule disassembly. These observations are similar to findings in pulmonary vascular endothelial cells [38]. As shown in Figure 7, the (TNF-α)-induced activation of p38 MAP kinase is markedly reduced upon pre-treatment with forskolin and rolipram, suggesting that the influence of cAMP could be due to regulation of the kinase itself. Consistent with this finding, a recent study in HeLa cells and fibroblasts showed that cAMP may act through the transcription factor CREB to bring about the expression of dynein light chain (DLC), which disrupted the formation of the MKK3/6-p38 complex upstream of p38 MAP kinase [54]. Increased cAMP in vascular endothelial cells has been shown to prevent thrombin-induced intracellular adhesion molecule (ICAM)-1 expression by inhibiting p38 MAP kinase activation [55]. These reports are in line with the effect of elevated cAMP on the inhibition of (TNF-α)-induced p38 MAP kinase activation, shown in Figure 7.

In conclusion, we have demonstrated for the first time that elevated cAMP opposes p38 MAP kinase activation by TNF-α, and thereby attenuates downstream events leading to barrier dysfunction in the corneal endothelium. Hence, elevation of cAMP can be a therapeutic strategy to overcome endothelial barrier dysfunction in response to TNF-α during corneal transplantation failure and/or uveitis.
